# Laser Pulpotomy–An Effective Alternative to Conventional Techniques: A 12 Months Clinicoradiographic Study

**DOI:** 10.5005/jp-journals-10005-1277

**Published:** 2015-04-28

**Authors:** Garima Gupta, Vivek Rana, Nikhil Srivastava, Preetika Chandna

**Affiliations:** Junior Resident, Department of Pedodontics and Preventive Dentistry Subharti Dental College, Meerut, Uttar Pradesh, India; Professor, Department of Pedodontics and Preventive Dentistry Subharti Dental College, Meerut, Uttar Pradesh, India; Principal and Head, Department of Pedodontics and Preventive Dentistry Subharti Dental College, Meerut, Uttar Pradesh, India; Reader, Department of Pedodontics and Preventive Dentistry Subharti Dental College, Meerut, Uttar Pradesh, India

**Keywords:** Electrosurgery, Ferric sulfate, Laser, Pulpotomy.

## Abstract

**Background:** Vital pulpotomy is a single-stage procedure of surgical amputation of the coronal portion of exposed vital pulp, usually as a means of preserving the vitality and function of the remaining radicular portion.

**Aims and objectives:** The aim of this study was to compare the clinical and radiographic success rates for ferric sulfate (FS), electrosurgery (ES) and laser pulpotomy in human primary molars.

**Materials and methods:** In a randomized clinical trial, 30 primary molars indicated for pulpotomy in children aged 4 to 10 years were treated using either a FS (10 teeth), ES technique (10 teeth) and laser (10 teeth). Following the pulpotomy, the teeth were evaluated for clinical and radiographic success at 3, 6, 9 and 12 months on the basis of the presence of pain, sinus, mobility, internal and external resorption, periapical radiolucency, calcification in the canal and bone loss.

**Statistical analysis:** The data were assessed with Chi-square test.

**Results:** After 12 months of follow-up, both clinical and radiographic success rates were 100% in the laser group but only 80% in both ES and FS groups. There was statistically significant difference between the success rates of three groups (p < 0.05).

**Conclusion:** Laser pulpotomy showed better clinical as well as radiographical results than ES and FS pulpotomy. Laser pulpotomy was also found superior in terms of operating time, patient cooperation, ease of use and pain. Although results of the study showed the failure rates for electrosurgical pulpotomy to be equal to those for FS pulpotomy, electrosurgical pulpotomy being a nonpharmacological technique considered more favorable. Further studies using larger sample size and longer evaluation periods are suggested.

**How to cite this article:** Gupta G, Rana V, Srivastava N, Chandna P. Laser Pulpotomy–An Effective Alternative to Conventional Techniques: A 12 Months Clinicoradiographic Study. Int J Clin Pediatr Dent 2015;8(1):18-21.

## INTRODUCTION

Vital pulpotomy is considered a one-stage procedure and is defined as ‘the surgical amputation of the coronal portion of an exposed vital pulp, usually as a means of preserving the vitality and function of the remaining radicular portion’.

Many pharmacotherapeutic agents/techniques have been used when performing pulpotomies in primary teeth. Formocresol (FC) was previously considered as gold standard^1^ for pulpotomy with high success rate of 97%.^2^ During the past six decades, it has been the most commonly used pulp-dressing material for pulpotomy of primary molars^3^ but, due to some significant disadvantages like cytotoxicity, potential mutagenicity^4^ and immune sensitization,^3^ clinicians prefer to use alternative methods which are more bio and tissue compatible.

Ferric sulfate (FS) is a coagulative and hemostatic agent. Many authors including Fei et al reported the application of FS in pulpotomized human primary molars with high clinical and radio graphic success. ^5^ No concerns about toxic or harmful effects of FS have been recorded in dental literature till date.^6^

Electrosurgical (ES) as a nonpharmacological pulpotomy technique has been well-documented and proven to have great merits. ^7^ Electrosurgery leads to good visualization and homeostasis and is less time consuming than the FC approach.^8^

A relatively newer nonpharmacotherapeutic method that has emerged is the use of laser in which the laser energy is able to overcome the histologic deficits there by accelerating the wound healing of the pulp and the expression of the lectins and collagens. Laser irradiation also enhances the formation of calcified nodules in human dental pulp fibroblasts, alkaline phosphatase activity, and the production of collagen and osteocalcin. Laser irradiation creates a superficial zone of coagulation necrosis that remained compatible with the underlying tissue and isolate the pulp from the subbase. The use of laser has also been suggested as an alternative, due to its hemostatic, antimicrobial, and cell-stimulating properties with only slight thermal alteration to the pulpal tissue.

The purpose of this clinical trial was to compare the clinical and radiographic success of FS, ES and laser pulpotomy used for pulpotomy of human primary molars requiring vital pulp therapy secondary to carious involvement.

## MATERIALS AND METHODS

Thirty patients between the age of 4 and 10 years were randomly selected from the Outpatient Department of Pediatric Dentistry of Subharti Dental College, Meerut. Informed consent was obtained from parents of all children participating in the study. Ethical approval was obtained from Institutional Ethical Committee. All patients were without any systemic illness. The patients having one or more deeply carious primary molars with following sign and symptoms were included in the study:

- No history of spontaneous or persistent pain.
- Lack of clinical evidence of pulpal degeneration, such as pain on percussion, history of swelling or sinus tracts.
- Restorable teeth following completion of procedure.
- Following pulpal amputation, hemostasis could be easily achievable within 5 minutes with a sterile moist cotton pellet.

Radiographic inclusion criteria were the following:

- Radiolucency approaching pulp.
- Minimum of 2/3rd of the root length should be remaining.
- No interradicular/periapical radiolucency.
- Absence of radiographic signs of internal and external resorption.

**Fig. 1:**
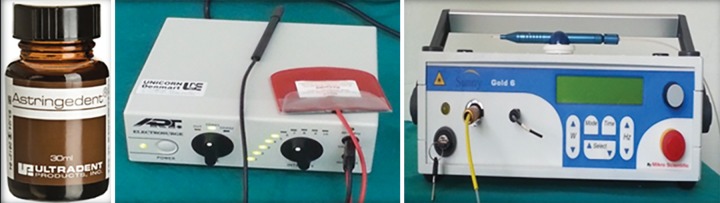
15.5% Ferric sulfate (Astringedent), electrosurgery unit (Bonart ART-E1) and diode laser (Sunny Gold)

All procedures were performed by one investigator. The patients included in the study were equally but randomly assigned to one of the three treatment groups: group 1: ferric sulfate (Astringedent), group 2: electrosur-gery (Bonart ART-E1) and group 3: laser (Sunny Gold) (Fig. 1). Once local anesthetic and quadrant rubber dam isolation was achieved, coronal pulp was amputated with low speed round bur and later with spoon excavator. The pulp chamber was flushed with 5 cc sterile saline and then dried with sterile cotton pellets. Hemostasis was achieved with moist cotton pellets placed under pressure for 5 minutes. In the FS group, sterile cotton pellet saturated with FS was placed in cleaned pulp chamber for 15 seconds.

In group 2 (ES), after hemostasis was achieved, an electrode tip of the ES unit T4 (fine wire) with 50 W power, 110 V ± 5% 50/60 Hz 92 VA and work frequency of 1.5 ~ 1.7 MHz ± 5% was used for the pulpotomy procedure. During the procedure, the electrode tip was positioned slightly above the pulp tissue but close enough for electrical arcing to occur (about 1 mm above the tissue). The current was applied for 1 to 2 seconds over each pulpal stump. This procedure was repeated up to three times on each pulpal orifice, until brown appearance was observed in the tissue.^8^

In group 3 (laser), after achieving hemostasis, the pulp was ablated to the level of the canal orifice using diode laser with 980 nm wavelength, 3 W of power and on continuous pulse mode. The laser energy of 4.0 J/cm^2^ was delivered through a 0·5 mm diameter opt ical fiber in contact with pulp tissue with the total energy of one spot, corresponding to 2 minutes and 31 seconds exposure. If additional ablation was required, subsequent multiple applications were administered. In all study groups, zinc oxide eugenol was placed directly on the radicular pulp stump and the teeth were restored with stainless steel crowns.

Patients were recalled after 3, 6, 9 and 12 months for clinical and radiographic evaluation. Statistical analysis was performed using Chi-square test.

## RESULTS AND DISCUSSION

The data obtained were tabulated at 3, 6, 9 and 12-month intervals both clinically and radiographically with respect to individual criteria.

**Fig. 2:**
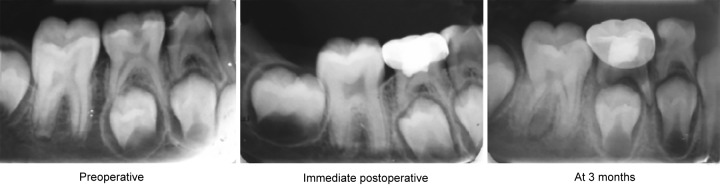
Radiographical failure of ferric sulfate (group 1)

In FS group, two patients showed pain (20% failure) after 3 months, X-ray also showed periapical and furcal radiolucency (Fig. 2) in the same two cases. The failure of pulpotomy treatment in primary molars could be attributed to the number of factors, one of which may be clinical errors in diagnosis and selection of the case; for example, chronically Inflamed radicular pulp was believed to be nonInflamed. Other possible reason could be the use of ZOE as sub-base which is in direct contact with the highly perfused environment of pulp and undergo hydrolysis of the zinc eugenolate to yield free eugenol. In FS, only the clot is the entity separating the eugenol from the vital tissue; therefore, ZOE may not bean ideal base for FS pulpotomies due to the Inflammatory tissue response.^9^

**Fig. 3:**
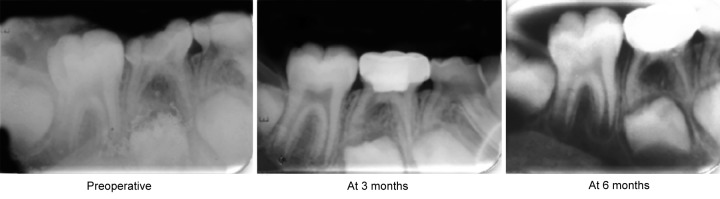
Radiographical failure of electrosurgery (group 2)

However, in laser and ES groups there was no clinical and radiographical failure at 3 months. Therefore, clinical and radiographical follow-up at 3 months shows 80% success rate in FS group whereas ES and laser groups had 100% success rate. The results obtained in this study were in accordance with Neamatollahi H^9^ et al, Gisoure EF^10^ and Huth KC^11^ et al but the study done by Sonmez D^12^ et al showed the success rate of 73.3%, with the possible explanation the differences in the applied techniques.

The comparison of clinical and radiographical outcomes of all the three groups at 6 months follow-up which in group II, two patients showed pain and internal resorption (Fig. 3) radiographically (20% failure). The reason for resorption could be explained as suggested by Shulman ER et al^13^ that the pulp tissue affected at some distance from the amputation site. The authors stated that it is likely that the main effect was not by cautery, but by the currents traveling down the canal. This finding might be expected with ES since electrical current follows the path of least resistance, which, in the case of a tooth is likely to be through the root canal. Based on this, the pulpotomy procedure in this tooth was considered a radiographic failure. However, in laser and FS groups, there was no clinical and radiographic failure. Thus, at 6 months follow-up, the clinical and radiographic success rates of FS, ES and laser groups were 80, 80 and 100% respectively. Thus, laser was better than FS and ES group clinically at 6 months (p < 0.05). Several studies including that of Dean JA, Mack RB^14^ and Mahmoud SH^15^ et al in the past have shown less than 100% success of electrosurgical pulpotomy which coincide with the results of present study. However, this rate was lower than that reported by Samad F^16^ et al and Mack RB and Jean DA^7^ with a success rate of 99.4% at 26 months follow-up.

**Fig. 4:**
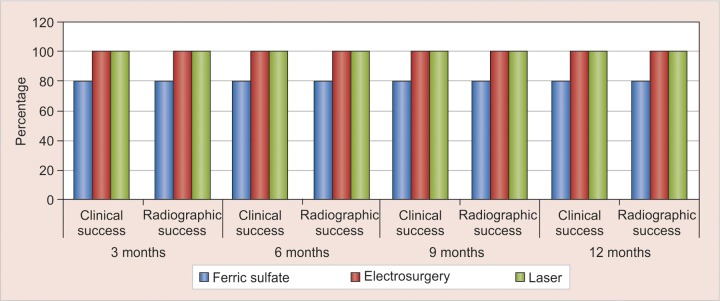
Overall clinical and radiographic follow-up at 3, 6, 9 and 12 months

No clinical as well as radiographic failure was noticed in any of the groups at 9 and 12 months. Therefore, at the end of 12 months success rates of groups I, II and III were 80, 80 and 100% respect ively and FS a nd ES had equivalent results, whereas laser had significantly better success rate of 100% (Fig. 4). The 100% clinical success of laser pulpotomy in our study could be attributed to its non-invasive and non-pharmaceutical nature of technique, efficient control of hemorrhage,^17^ decontamination^18^ and sterilization^17^ effect simultaneously with preservation of the radicular pulp,^17^ and faster pulpal wound healing that did not affect either the Inflammatory function of monocytes and endothelial cells or the adhesion of endothelial cells.^17^

Through in the present study, an attempt was made to compare the three approaches available for pulpotomy. The use of FS (preservative) in this study proved to be less acceptable agent for pulpotomy. Electrosurgical pulpotomy (devitalization, nonpharmacotherapeutic) provided good overall success. Additionally, the absence of any pharmacological side-effects with ES, justified its use as a safe and nontoxic pulpotomy agent. One of the most intriguing aspects of this study was the use of laser (devitalization, nonpharmacotherapeutic) which showed an acceptable success in this study.

## CONCLUSION

Laser pulpotomy showed 100% success both clinically and radiographically at 12 months interval. Electrosurgery and ferric sulfate, though both were found to be successful in 80% cases, ES was considered better due to its advantage of being nonpharmacological. However, further studies are needed to evaluate the histological effects of these methods as well as compare these methods to pulpotomy with new bioregenerative materials.
